# Arsenic Sulfide Promotes Apoptosis in Retinoid Acid Resistant Human Acute Promyelocytic Leukemic NB4-R1 Cells through Downregulation of SET Protein

**DOI:** 10.1371/journal.pone.0083184

**Published:** 2014-01-13

**Authors:** Yuwang Tian, Yanfeng Liu, Pengcheng He, Feng Liu, Naicen Zhou, Xiaoyan Cheng, Lili Shi, Huachao Zhu, Jing Zhao, Yuan Wang, Mei Zhang

**Affiliations:** 1 Department of Pathology, General Hospital of Beijing Military Area of PLA, Beijing, China; 2 Department of Hematology, The First Affiliated Hospital, School of Medicine, Xi'an Jiaotong University, Xi'an, Shaanxi, China; German Cancer Research Center, Germany

## Abstract

Tetra-arsenic tetra-sulfide (As_4_S_4_) is an arsenic compound with anti-tumor activity, especially in acute promyelocytic leukemia (APL) that are resistant to retinoic acid (RA). Although recent studies revealed that the therapeutic action of As_4_S_4_ is closely associated with the induction of cellular apoptosis, the exact molecular mechanism of action of As_4_S_4_ in RA-resistant APL remains to be clarified. In this study, we found that As_4_S_4_-induced apoptosis was accompanied by reduced mRNA and protein expression of SET gene in RA-resistant NB4-R1 cells. Moreover, RNAi knockdown of SET gene further promoted As_4_S_4_-induced apoptosis, while SET over-expression inhibited it, suggesting that As_4_S_4_ induces apoptosis through the reduction of SET protein in NB4-R1 cells. We also demonstrated that the knockdown of SET gene resulted in the upregulation of protein phosphatase 2 (PP2A) expression and the downregulation of promyelocytic leukemia and retinoic acid receptor α fusion gene (PML-RARα) expression, which were enhanced by As_4_S_4_ treatments. By contrast, over-expression of SET gene resulted in PP2A downregulation and PML-RARα upregulation, which were abolished by As_4_S_4_ pretreatment. Since PP2A is a pro-apoptotic factor and PMLRARα is an anti-apoptotic factor, our results suggest that As_4_S_4_-induced apoptosis in NB4-R1 cells is through the downregulation of SET protein expression, which in turn increases PP2A and reduces PML-RARα expressions to lead to cell apoptosis.

## Introduction

Acute promyelocytic leukemia (APL), also known as acute progranulocytic leukemia, is a subtype of acute myelogenous leukemia (AML). APL is characterized by a severe risk of early hemorrhagic death caused by a combination of disseminated intravascular coagulation (DIC) and hyperfibrinolysis [1], [2]. APL is also a morphological M3 subtype of AML and is characterized cytogenetically by a reciprocal translocation between chromosomes 15 and 17, which results in the fusion gene of promyelocytic leukemia (PML) gene and retinoic acid receptor α (RAR α) gene [1], [3]. This fusion protein, PML-RARα, binds with enhanced affinity to sites on the cellular DNA and enhances interaction of nuclear co-repressor (NCOR) molecule and histone deacetylase (HDAC), thus blocking transcription, differentiation of granulocytes, and inhibition of apoptosis [4], [5]. All *trans* retinoic acid (ATRA) in combination with anthracycline-based chemotherapy is the standard treatment modality for APL and is able to induce complete remission (CR) in most of the patients with APL through *in vivo* differentiation of APL blasts, resulting in cure rates exceeding 80% [6], [7]. More recently, arsenic trioxide (As_2_O_3_ or ATO), with or without ATRA, has shown high efficacy and reduced hematologic toxicity in APL treatment and has been approved for the treatment of relapsed patients both in the United States and Europe [8].

Approximately 75% patients with APL achieved CR after receiving traditional chemotherapy, which includes daunorubicin (DNR) or 4-(9-acridinylamino) methanesulfan-m-anisidide (AMSA) in combination with arabinosylcytosine (Ara-C) and 6-thioguanine (TG) [9], however, traditional chemotherapy can lead to early hemorrhagic death due to abnormalities of blood coagulation that occurs in most of the patients at diagnosis. Although ATRA is considered to be a relatively safe drug and more than 90% APL patients were reported to achieve CR [10], [11], drug resistances and side effects such as retinoic acid syndrome and psedudotumor cerebri can occur when using ATRA (PC) [12], [13]. Therefore, development of new drugs with higher efficacy and lower toxicity is still needed for APL treatment. Despite the well known toxicity of arsenic, As_2_O_3_ is an efficacious agent for the treatment of APL in either primary or relapsed patients [14], [15], [16]. Tetra-arsenic tetra-sulfide (As_4_S_4_) is another arsenic compound with anti-tumor activity, especially on hematological malignancies. Moreover, multi-dose oral As_4_S_4_ is safe and relatively well tolerated in APL patients [17]. Lu et al observed that oral As_4_S_4_ was highly effective and safe in both remission induction and maintenance therapy in 129 patients with APL, regardless of disease stages [18]. In addition, As_4_S_4_ also has potential clinical applications when combined with imatinib in the treatment of chronic myelogenous leukemia (CML) [19]. The molecular mechanisms for the anti-tumor action of As_4_S_4_ were shown to be through the induction of apoptosis [19], [20] and/or through the redistribution of PML-RARα protein in leukemic cells from APL patients [21].

Our previous study demonstrated the induction ability of cellular apoptosis of As_4_S_4_ in RA-resistant cells by using a serial *in vitro* assays [22]. Moreover, we identified several As_4_S_4_ targeted proteins, such as SET/template-activating factor (TAF-1β), RPP2, and PHB by using the high-resolution two-dimensional electrophoresis system and mass spectrometry [22]. In the current study, we further investigated the role of the oncoprotein SET/TAF-1β in inducing apoptosis by As_4_S_4_ in RA-resistant human APL NB4-R1 cells.

## Materials and Methods

### Cell culture and reagents

The NB4-R1 APL-derived cell line is a RA-resistant promyelocytic cell line, which was a gift from the School of Medicine, Shanghai Jiao Tong University [22]; it was cultured in RPMI 1640 (GIBCO, BRL, USA) supplemented with 10% heat inactivated fetal bovine serum in a humidified incubator containing 5% CO2 and 95% air at 37°C. As_4_S_4_ (Xi'an Traditional Chinese Drug Company, China) stock solution was prepared by dissolving in 1.0M NaOH.

### MTT assay

MTT assay was used to test the cytotoxic effect of As_4_S_4_ (2–50 µmol/L) on NB4-R1 cells. Control and treated cells were cultured in sterile 96-well plates at an optimal cell density of 5×10^5^/ml per well and were incubated at 37°C in 5% CO^2^ incubator for 24 h, 48 h and 72 h respectively (n = 6). Then, they were assayed for cell viability using the colorimetric MTT assay as described previously [22]. A growth curve was drawn according to MTT colorimetry. Percentage growth inhibition was equal to [1−(OD of treated/OD of control)]×100%. IC50 (the concentration inhibiting 50% of in vitro cell growth) was calculated by SPSS 15.0.

### Transmission electron microscopy

NB4-R1 cells treated with As_4_S_4_ (25 µmol/L, 24 h, 48 h) or control were harvested and fixed in phosphate-buffered 2.5% glutaraldehyde and 1% osmium tetroxide, followed by dehydration through graded ethanol. The samples were then embedded in Epon 812, thin-sectioned, and stained with uranium acetate and plumbum citrate. The slides were subsequently examined under a JEM-100SX electron microscope (JEOL Company, Japan).

### Identification of differentially expressed proteins by MS and MS/MS

Frozen cell samples prepared from NB4-R1 untreated (R0) and samples treated with 25 µmol/L As_4_S_4_ for 24 h (R24) and 48 h (R48) cells, were dissolved in lysis buffer containing 40 mM Tris base, 8M urea, 2M thiourea, 4% (w v^−1^) 3-[(3-cholamidopropyl) dimethylammonio]-1-propanesulfonate (CHAPS), 1% (w v^−1^) dithiothereitol (DTT), 1 mM EDTA and 1× protease inhibitor cocktail (Roche Diagnostic, Indianapolis, IN), freezing and thawing for three times in liquid nitrogen. After centrifugation at 14,000×g (Sigma-Aldrich, St. Louis, MO, USA) for 30 min at 4°C, the supernatant was used as two-dimensional electrophoresis (2-DE) sample, and the protein concentration was determined by the Bradford method with a commercial Bradford reagent (Bio-Rad Laboratories, Hercules, CA). 2-DE was performed as follows. 140 µg of proteins for analytical gels or 1.4 mg of proteins for micropreparative gels were briefly diluted to 350 µl with rehydration solution [8M urea, 2% (w v^−1^) CHAPS, 60 mM DTT, and 0.8% immobilized pH gradient (IPG) buffer (Amersham Pharmacia Biotech, Piscataway, NJ)] and applied onto IPG gel grove. The total voltage-time was 20–22 kVh. 18 cm (pH 3–10) not linear immobilized pH gradient Drystrip (Amersham Pharmacia Biotech). The strips were rehydrated for 11 h at 20°C. The proteins were then focused on the IPGphor system (Amersham Pharmacia Biotech) according to the manufacturer's protocol. The strips were then equilibrated for 15 min in a solution containing 6M urea, 2% (w v^−1^) SDS, 20 mM DTT, 30% (w v^−1^) glycerol and 50 mM Tris–HCl (pH 8.8). A second equilibration step was also carried out for 15 min in the same solution but DTT was replaced by 100 mM iodoacetamide. Separation in the second-dimensional electrophoresis was carried out in the PROTEAN α xi Cell (Bio-Rad company, Richmond, CA, USA) with a 13% SDS-polyacrylamide gel without a stacking gel at a constant current of 20 mA/gel for the initial 40 min and 30 mA/gel thereafter until the bromphenol blue dye marker reached the bottom of the gel. The samples from the same treatment were run at least two times in order to determine the variability.

Silver nitrate staining and Coomassie Brilliant Blue R-250 (0.05% Brilliant Blue) were used for the analytical and micropreparative gels, respectively. 2-DE images were analyzed with an ImageScanner (Amersham Pharmacia Biotech). Spot detection, quantification, and alignment were performed with the ImageMasterTM 2D Platinum software (Amersham Pharmacia Biotech). Intensity levels were normalized between gels by expressing the intensity of each spot in a gel as a proportion of the total protein intensity detected for the entire gel. Spot relative volumes were normalized for every gel [(spot volume)/˙(spot volumes)×10^4^] to correct for subtle variation in protein loading and gel staining between the gels to be compared.

After matching the micropreparative gel image with the analytical image, the in-gel digestion was performed. 0.5–1 µl sample solution and equal volume of the saturated matrix solution were mixed and applied onto the target plate. All mass spectra of MALDI-TOF-MS were obtained on a Bruker REFLEX III MALDI-TOF-MS (Bruker-Franzen, Bremen, Germany) in positive ion mode at an accelerating voltage of 20 kV. Monoisotopic peptide masses, used to search the database, allowed a peptide mass accuracy of 0.3 Da and one partial cleavage. Oxidation of methionine and carbamidomethyl modification of cysteine were also considered. The obtained PMF were used to search through the SWISS-PROT and NCBInr database by the Mascot search engine.

### RNA Extraction and Real-time PCR

RNA extracted from NB4-R1 cells (RNeasy Mini Kit; Qiagen) were used to synthesize first-strand cDNA from total RNA (SuperScript First-Strand Synthesis System; Invitrogen, Carlsbad, CA). Real Time-PCR primers: human SET forward: 5′-aaatataacaaacctccgccaacc-3′ and reverse: 5′-cagtgcctcttcatcttcctc-3′; The GAPDH was used as internal control using the following primers: forward: 5′-tgcaccaccaatgcttag-3′ and reverse: 5′-ggatgcagggatgatgttc-3′. The real-time PCR reaction containing 10 ng cDNA, 1× SybrGreen Supermix, 0.25 mmol/L forward, and reverse primers was carried out following three-step amplification protocol in QuantiTect; ABI PRISM 7700 machine, and the melt curves were analyzed in iQ5 Real-time PCR Detection System (Bio-Rad). The standard curve was prepared with amplified cDNA using 5-fold dilution series of 100 to 0.16 ng cDNA per reaction. Relative gene expression was calculated using the glyceraldehyde-3-phosphate dehydrogenase expression value.

### Western blotting (WB) assay

NB4-R1 cells were washed once with ice-cold PBS and disrupted by homogenization in RIPA buffer (Sigma). Protein concentration was determined by BCA kit. Protein expression was analyzed by Western blot (20 µg/lane) using anti-SET specific antibody (1∶ 1000). Level of GAPDH protein was used as loading control. Protein-bands were detected using Super Signal West Pico Chemiluminescent Substrate (Pierce, Rockford, IL) and exposed on Kodak X-OMAT film (Kodak). For WB assay, in each experiment, we did three times. Densitometric analysis of bands was carried-out with Quantity One 4.6.2 software.

### Plasmid construction and lentivirus production

pHelper 1.0, pHelper 2.0 and pGCSIL-GFP plasmids were purchased from Shanghai GeneChem Co. Ltd. (Shanghai, China). To construct the recombinant vector, RNAi stem-loop DNA oligos containing the target sequences (GCGATTGAACACATTGATG) in the region of SET gene were chemically synthesized, annealed, and cloned into the *Age*I/*Eco*RI-digested pGCSIL-GFP, thus generating the lentiviral vector pGCSIL-SET. A control shRNA that targets none of human genes was also designed and cloned into pGCSIL-GFP to obtain the control vector pGCSIL-GFP-Mock. In these two vectors, the expression of shRNA was controlled by a U6 promoter, and the expression of green fluorescent protein (GFP) was controlled by the CMV promoter, which was used as a reporter gene to detect the transfection efficiency of viral packaging and infection. Recombinant lentiviruses were generated by co-transfection of 293T cells with 20 µg pGCIL-SET-GFP or pGCIL-GFP-Mock, and packaging vectors (15 µg pHelper1.0, 10 µg pHelper2.0) using 100 µl of Lipofectamine 2000™ reagent according to the manufacturer's instructions (Invitrogen, Grand Island, NY). The viral supernatant was harvested 48 h after transfection, passed through 0.45 µm filters, and concentrated. The viral titer was determined by infecting 293T cells with serial dilutions of concentrated lentivirus, and then determining the GFP expression of infected cells by fluorescence microscopy 24 h after infection. Therefore, the titer is expressed as “transduction unit (TU)/ml”. A scramble siRNA sequence (5′-UUCUCCGAACGUGUCACGU-3′) was used to generate the non-silence control plasmid and lentivirus that were designated pGCL-NC.

### RNA Knockdown assay

Semi-confluent NB4-R1 cells were cultured in 6-well plates and transfected with pGCL-NC or pGCL-SET. After 24 h or 48 h of transfection, the percent of GFP-expressing was counted using fluorescence microscopy. The total RNA and protein were prepared from the cells with transfection efficiency over 70%.

### Construction of plasmids for SET over-expression

SET cDNA was obtained by PCR and cloned into the pEGFP-N1-3FLAG, which was designated as pEGFP-N1-SET. PCR used the Expand Long Template PCR System (Roche Applied Science) that contains thermostable Tag DNA polymerase and Tgo DNA polymerase), a thermostable DNA polymerase with proofreading activity. The PCR product was digested with XhoI and Kpn I, and then cloned into pEGFP-N1-3FLAG to generate the recombinant plasmid pEGFP-N1-SET that encodes SET gene. The correct orientation and sequences of SET cDNA in pEGFP-N1-SET were verified by DNA sequence analysis. Semi-confluent NB4-R1 cells cultured in 6-well plates were transfected with pEGFP-N1-3FLAG or pEGFP-N1-SET. After 24 h or 48 h of transfection, the GFP-expressing cells were counted under fluorescence microscopy. The total RNA and protein were prepared from the cells with transfection efficiency over 70%.

### Annexin V-FITC/PI

Flow cytometric analysis using Annexin V FITC (Sigma, USA) and propidium iodide (PI, Sigma, USA) were used to analyze the apoptotic NB4-R1 cells after As_4_S_4_ treatment for 24 and 48 h, respectively. NB4-R1 cells were briefly washed twice with cold PBS at 4°C and re-suspended in 1× binding buffer. Annexin V-FITC (25 µg/mL) and PI (5 µg/mL) were added to the cell suspension. After 15 min of incubation in dark at room temperature, analysis was performed by flow cytometer (Becton Dickinson FACS caliber double laser flow cytometer) immediately. Flow cytometric reading was taken using 488 nm excitation and band pass filters of 530/30 nm (for FITC detection) and 585/42 nm (for PI detection). Data analysis was performed by CellQuest software program.

### Statistical analysis

SPSS 15.0 software was used for data analysis. All experiments were repeated at least three times with different cell preparations. The data was expressed as mean ± standard deviation. ANOVA was used for multi-group comparison. The difference between two groups was determined by a t-test. P values less than 0.05 were considered statistically significant.

## Results

### As_4_S_4_ inhibits the proliferation of NB4-R1 cells

**Figure 1:**
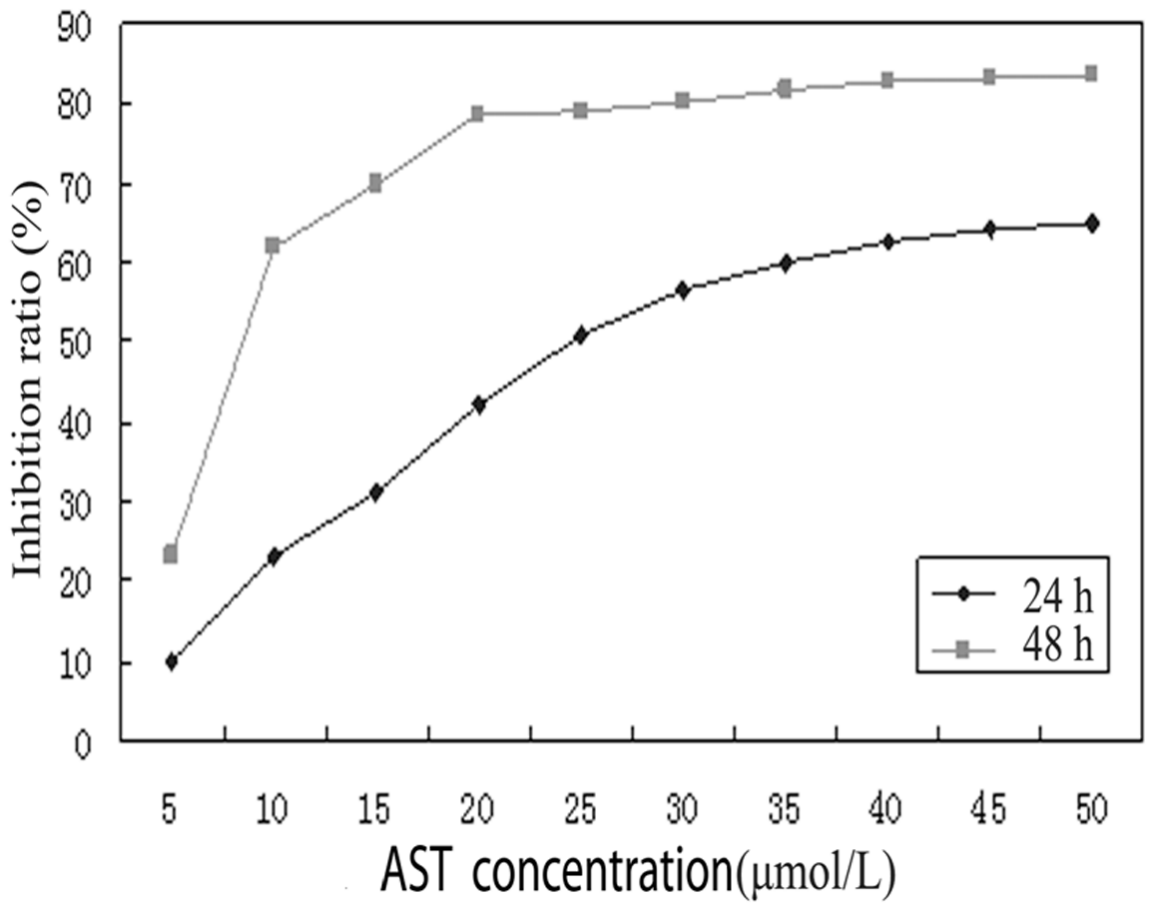
Comparative cytotoxicity of As_4_S_4_ in NB4-R1 cells by the MTT assay. Dose- and time-dependent curve of inhibition rate of As_4_S_4_ on NB4-R1 cells by the MTT assay. Data were presented as the means±SD of three independent experiments performed in quintuplicate. Each value represents the mean ± SD of triplicate experiments. MTT: (3-[4,5-dimethylthiazol-2-yl]-2,5 diphenyl tetrazolium bromide): SD: standard deviation.

We first determined the inhibitory effect of As_4_S_4_ on the proliferation of NB4-R1 cells. As shown in **Figure 1**, the inhibitory effect of As_4_S_4_ on NB4-R1 cell proliferation was in a dose- and time-dependent manner. Under the treatment with different concentrations of As_4_S_4_ (2–50 µmol/L), the inhibition rates ranged from 9. 97±2.35% (24 h) to 80.82±4.21% (48 h), with IC50 about 24.18±0.19 µmol/L at 24 h, and 9.50±0.13 µmol/L at 48 h after treatment. We therefore chose 25 µmol/L, the IC50 at 24 h, as doses of As_4_S_4_ for the following experiments.

### The morphological changes of As_4_S_4_ on NB4-R1 cells

**Figure 2:**
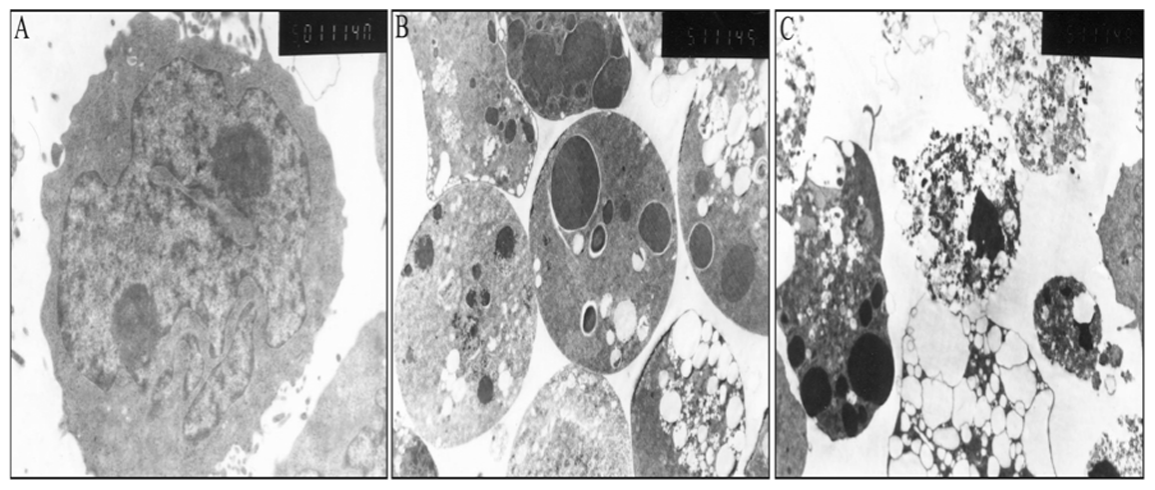
Ultrastructural changes in As_4_S_4_ treated NB4-R1 cells by TEM. Ultrastructural changes in untreated and As_4_S_4_ treated NB4-R1 cells by transmission electron microscopy (TEM) (original magnification: ×5000): (A) Untreated control cells showed intact nuclei and membrane. (B) As_4_S_4_ treatment for 24 h showed exhibited vacuolization, cytoplasm and chromatin condensation with intact membrane and nuclei. (C) As_4_S_4_ treatment for 48 h showed nuclear fragmentation and apoptotic bodies formation.

To determine whether the inhibition of cell proliferation by MTT assay after treatment with As_4_S_4_ was attributed to the induction of cellular apoptosis, ultrastructural characteristics of the cells were evaluated by transmission electron microscopy (TEM). The transmission images of untreated control cells showed intact nuclei and membrane (**Figure 2A**), while 25 µmol/L As_4_S_4_ treated NB4-R1 cells for 24 h exhibited vacuolization, chromatin, and cytoplasmic condensation with intact nuclei and membrane (**Figure 2B**), followed by prominent nuclear fragmentation and marginization of fragmented nuclei towards the membrane, and formation of apoptotic bodies after exposure to As_4_S_4_ for 48 h (**Figure 2C**). These morphological features of cells indicated that As_4_S_4_ could induce apoptosis in NB4-R1 cells.

### Proteome analysis of NB4-R1 cells treated with and without As_4_S_4_

**Figure 3:**
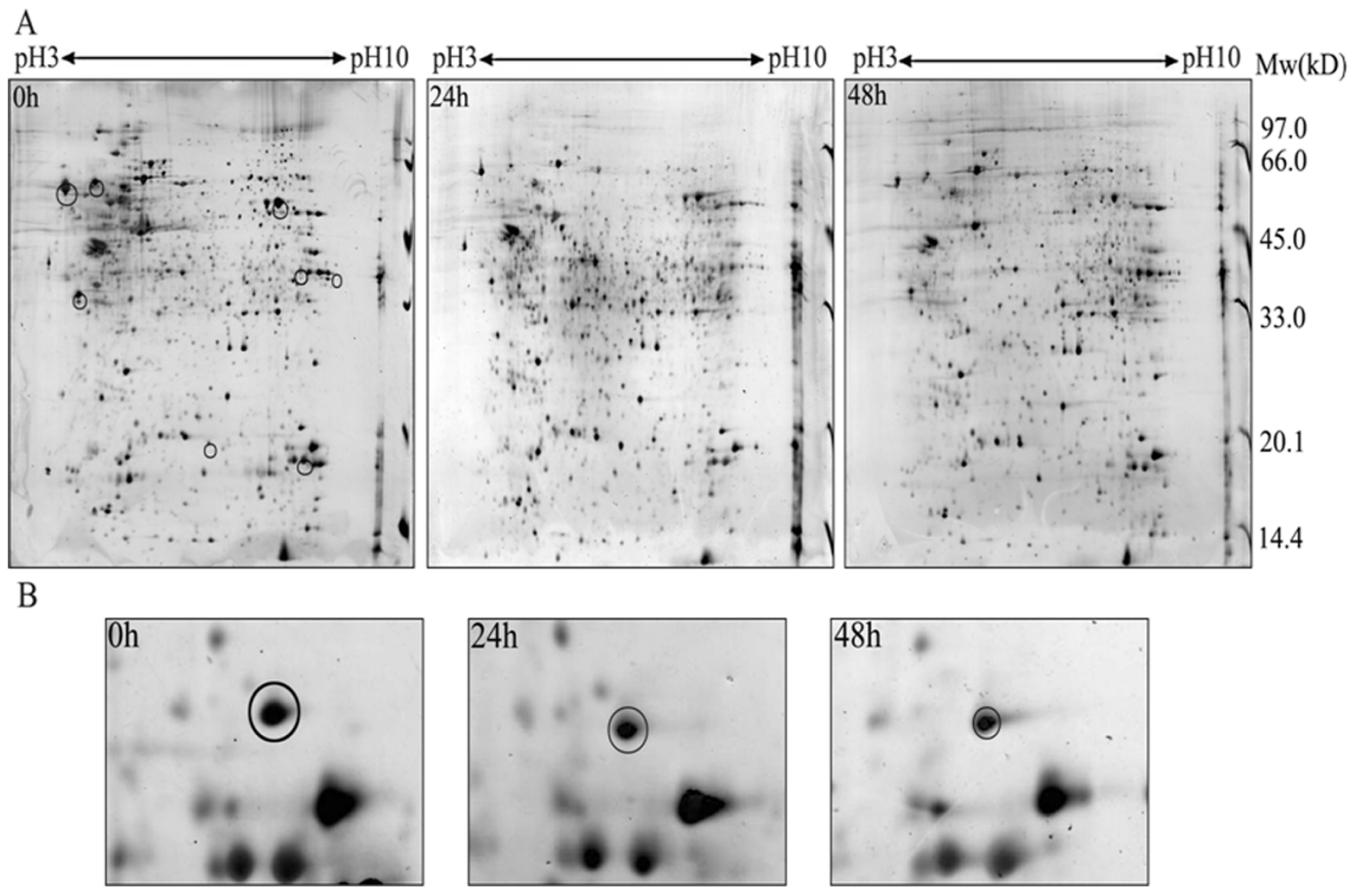
Proteomic comparison between untreated and As_4_S_4_ treated NB4-R1 cells using 2-DE and MALDI-TOF-MS. (A) Untreated, As_4_S_4_ treated for 24 h and 48 h NB4-R1 cells; (B) Enlarged regions of SET expressed representative protein spots in 0, 24, and 48 h map. 2-DE: two-dimensional electrophoresis.

In order to identify the differentially expressed proteins that are associated with As_4_S_4_-induced aopotosis in NB4-R1 cells, we performed 2-DE on the cell samples prepared from NB4-R1 that were untreated (R0), treated with 25 µmol/L As_4_S_4_ for 24 h (R24) or 48 h (R48). All maps from untreated and treated cells showed high resolution and good repeatability in their protein expression patterns (**Figure 3A**). Averagely 231±7, 219±9 and 196±7 spots could be detected on silver nitrate staining gel of R0, R24 and R48 respectively by the autodetect spots menu of ImageMasterTM software. Matching rate of the spots between R24 gel and R0 gel was 79.94%. Matching rate of the spots between R48 gel and R0 gel was 69.33%. To accurately quantify the changes of expression level of the same protein spot, relative amount (volume %) of each spot, which was the percent of all the spots in a gel, was represented as the expression level of a protein spot. The normalized data obtained by ImageMasterTM 2D Platinum software was statistically analyzed with Student's t-test. Combining above matching analysis with artificial comparison, 8 spots with significant expressing changes more than 3-fold were selected for mass spectrometry, including 2 protein spots up-regulated after exposing As_4_S_4_ for 24 h and 3 protein spots up-regulated after exposing As_4_S_4_ for 48 h. There were 2 protein spots which were down-regulated after exposing As_4_S_4_ for 24 h and were not detected in R48 gel. Only 1 protein spot consistently down-regulated after exposing As_4_S_4_ for 24 h and 48 h, and it was finally identified as protein SET (Table 1) (**Figure 3B**).

**Table 1:** Differentially expressed proteins in As_4_S_4_-treated/untreated NB4-R1 cells identified by MALDI-TOF-MS.

Spot	Protein name	SWISS-PROT accession no.	NCBI accession no.	Mass weight (Da)	P*I*	Sequence coverage(%)	Function
R0-1	SET	Q01105	gi:170763500	33,489	4.23	30	Oncoprotein
R0-2	α-tubulin	P07437	gi:338695	49,671	4.78	53	Microtubule assembly
R0-3	Poly(C)-binding protein 1 (PCBP1)	Q15365	gi:222352151	37,498	6.66	54	poly (C)-binding ability
R0-4	ACTB	P60709	gi:117167827	41737	5.29	17	Cell motility
R48-5	High-mobility group box 1 protein (HMGB1)	P09429	gi:48145843	24,894	5.62	33	Gene expression regulation
R48-6	Phosphoglycerate mutase (PGM)	P36871	gi:189926	61,449	6.30	51	Glucose breakdown and synthesis
R48-8	Rho GDP dissociation inhibitor 2 (RhoGDI2)	P52566	gi:56676393	22,988	5.10	54	Signal transduction
R48-9	Heat shock protein 90 kDa (HSP90)	P08107	gi:194248072	70.052	5.48	19	Molecular chaperone

### Down-regulation of SET protein and mRNA expression in NB4-R1 cells treated with As_4_S_4_

**Figure 4:**
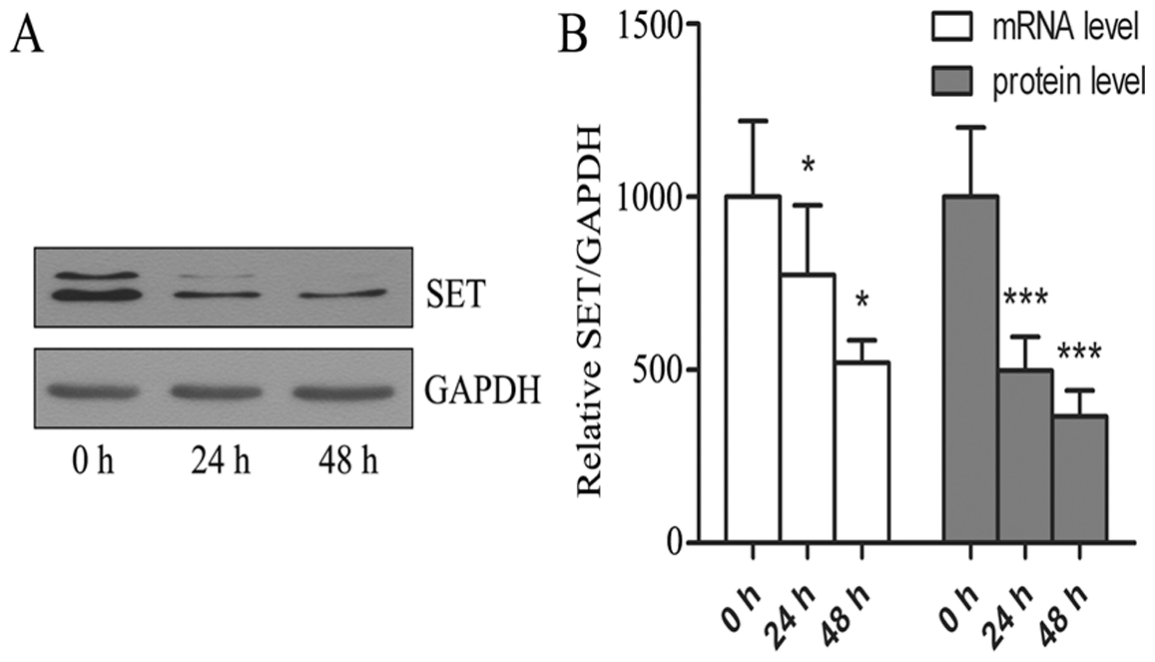
Down-regulation of SET protein and mRNA expression in NB4-R1 cells treated with As_4_S_4_. (A) SET protein expression in As_4_S_4_ treated NB4-R1 cells, 24 h and 48 h. (B) SET mRNA expression in As_4_S_4_ treated NB4-R1 cells, 24 and 48 hrs.

To confirm the reliability of mass spectrometry proteomic data, we quantitatively determined SET protein and mRNA expression in NB4-R1 cells treated with As_4_S_4_. As seen from **Figure 4A**, SET protein expression was down-regulated in NB4-R1 cells treated with 25 µmol/L As_4_S_4_ by 47.2±4.22% (p<0.01) at 24 h and 58.4±5.16% (p<0.01) at 48 h, respectively, in comparison with untreated cells. Meanwhile, SET mRNA expression was also down-regulated in NB4-R1 cells treated with 25 µmol/L As_4_S_4_ by 23.6%±3.52% (p<0.05) at 24 h and 47.9%±5.07% (p<0.05) at 48 h, respectively (**Figure 4B**).

### Induction of apoptosis by As_4_S_4_ is through the downregulation of SET expression in NB4-R1 cells

**Figure 5:**
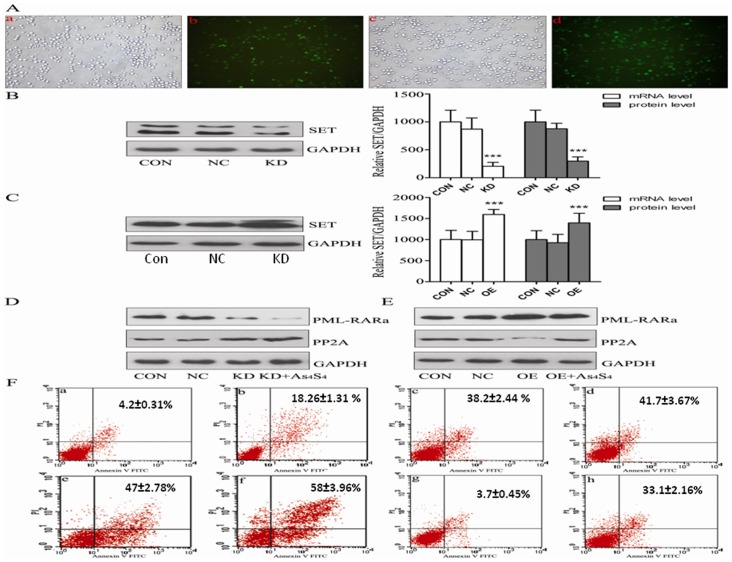
SET knockdown by siRNA or over-expression in inducing apoptosis by As_4_S_4_ in NB4-R1 cells using Annexin V-FITC/PI double staining and flow cytometry analysis. (A) GFP detection of transfection efficiency through fluorescence microscopy. SET knockdown by shRNA (a, b), SET over-expression (c, d). (B) SET protein down-regulated by shRNA-SET (C) SET protein over-expression by SET expression plasmid. (D) PML-RARα and PP2A protein expression in cells by shRNA-SET. (E) PML-RARα and PP2A protein expression in cells by using SET expression plasmid. (F) Annexin V-FITC/PI double staining and flow cytometry analysis.

In order to investigate whether or not SET is the key mediator of As_4_S_4_ induced apoptosis in NB4-R1 cells, we constructed two plasmids, SET RNAi that was able to silence SET and SET expression plasmid that was able to over-express SET. The transfection efficiencies of both plasmids were close to 75% (**Figure 5A**). Real-time PCR and Western blot analysis revealed that SET RNAi was able to inhibit 80% SET expression (**Figure 5B**), while SET overexpression plasmid was able to increase the expression of SET by 65% (**Figure 5C**).

We next investigated whether or not the knockdown and over-expression of SET gene had any effect on As_4_S_4_-induced apoptosis in NB4-R1 cells. By using Annexin V-FITC/PI double staining and flow cytometry analysis, we further confirmed that there were only 4.2±0.31% apoptotic cells in negative control NB4-R1 cells, but 18.26±1.31%, 38.2±2.44% apoptotic cells in NB4-R1 cells treated with As_4_S_4_ for 24 and 48 h, respectively. As expected, knockdown of SET gene by siRNA increased the apoptotic cells in NB4-R1 cells, which was 41.7±3.67%. Compared to As_4_S_4_ treatment alone (Figure 5F b, c), and As_4_S_4_ plus SET RNAi significantly enhanced apoptosis (Figure 5F f), which was up to 58±3.96%. By contrast, over-expression of SET gene plus As_4_S_4_ treatment significantly decreased apoptosis in NB4-R1 cells to a level that was not significantly different from the negative control, indicating that SET gene overexpression abolished As_4_S_4_-induced cell apoptosis (**Figure 5F** **h**). These results suggest that As_4_S_4_-induced cell apoptosis might be through the downregulation of SET expression.

### As_4_S_4_ alters SET-regulated PP2A and PML-RARα expressions in NB4-R1 cells

PP2A is a pro-apoptotic protein, and SET is its natural inhibitor. PML-RARα is an anti-apoptotic fusion protein, which can be enhanced by SET. As shown in Figure 5C, knockdown of SET by RNAi enhanced the expression of PP2A and reduced the expression of PML-RARα. In the presence of As_4_S_4_, the SET RNAi-induced upregulaton of PP2A was further increased, and the PML-RARα downregulation was further reduced. Also in the presence of As_4_S_4_, the SET RNAi-induced uppregulation of PP2A was further increased, and the PML-RARα downregulation was further reduced. In the presence of As_4_S_4_, the downregulated PP2A and upregulated PML-RARα expressions induced by SET overexpression were restored. These results suggest that As_4_S_4_ may induce apoptosis through the downregulation of SET protein expression, thereby increases PP2A expression and reduce PML-RARα expression, leading to the apoptosis of NB4-R1 cells.

## Discussion

Clinical use of As_4_S_4_ in the APL treatment can be either in composite formulas as a standard practice of traditional Chinese medicine or as a single agent [23], [24]. Current studies have shown that As_4_S_4_, as a new oral arsenic formulation, is highly effective and safe in the treatment of newly diagnosed APL patients in both remission induction and maintenance therapy regardless of disease stage, and more importantly in relapsed/refractory APL patients with ATRA resistance [18], [25]. Compared to As_2_O_3_, As_4_S_4_ is generally well tolerated with moderate side effects and possesses the biologic property of less toxical and adverse reaction [18]. Although recent studies revealed that the therapeutic action of As_4_S_4_ is closely associated with the induction of cellular apoptosis [19], [26], [27], [28], the definitive molecular mechanism of action of As_4_S_4_ in APL therapy still remains unknown. In the present study, As_4_S_4_ was further confirmed to inhibit the growth of RA-resistant NB4-R1 cells in a time- and dose-dependent manner. The increased number of apoptotic cells observed in NB4-R1 by electron microscopic; flow cytometric analyses confirmed that As_4_S_4_ inhibited tumor cell growth via inducing apoptosis. By performing 2-DE of cell lysates from As_4_S_4_ treated versus untreated cells and MS or MS/MS analysis, we identified 8 proteins that were significantly changed (more than 3-fold changes) in NB4-R1 cells for the first time. We selected one of proteins, SET for further study and found that SET is the key mediator of As_4_S_4_ induced apoptosis in NB4-R1 cells. We found that As_4_S_4_-induced apoptosis in NB4-R1 cells was significantly enhanced by knockdown of SET gene but abolished by overexpression of SET, indicating that As_4_S_4_-induced cell apoptosis might be through the downregulation of SET expression. We further demonstrated that knockdown of SET by RNAi enhanced PP2A and reduced PML-RARα expressions. In contrast, the overexpression of SET inhibited PP2A expression enhanced PML-RARα expression. Also in the presence of As_4_S_4_, the SET RNAi-induced uppregulation of PP2A was further increased, and the PML-RARα downregulation was further reduced. In the presence of As_4_S_4_, the downregulated PP2A and upregulated PML-RARα expressions induced by SET overexpression were restored. Because PP2A is a pro-apoptotic protein and SET is its natural inhibitor, our results suggest that As_4_S_4_ may induce apoptosis through the downregulation of SET protein expression, thereby increases PP2A expression and reduce PML-RARα expression, leading to the apoptosis of NB4-R1 cells.

SET, also called I2PP2A or TAF-1β, is an inhibitor of histone acetyltransferase, which inhibits active demethylation of DNA, integrates DNA methylation, and transcriptional silencing [29]. As an intracellular inhibitor of serine/threonine phosphatase PP2A [30], SET was first identified in acute non-lymphocytic leukemia as part of the SETCAN (nucleopoin Nup214) fusion protein resulting from a gene translocation. Phosphorylation of SET protein at ser171 by protein kinase D2 diminishes its inhibitory effect on PP2A[31]. It was reported that SET protein is leukemogenic and is the natural inhibitor of PP2A, which destroys the activity of PP2A [32]. It is also an inhibitor of the tumor suppressor NM23-H1 [32], and it was reported to be associated with the oncoprotein MLL (mixed lineage leukemia also termed ALL1, HRX) in AML [33]. High levels of SET have been detected in a number of different human malignancies, including cancers from uterus, colon, stomach, and rectum [29], ovarian tumor [34], Wilms' tumor and leukemia [35], [36], thus implying an oncogenic role of SET in tumorigenesis. Moreover, SET overexpression is associated with a poor outcome in AML [37]. Therefore, SET overexpression may also be critical for tumorigenesis of APL and RA-resistance.

Besides rapid reduction of SET expression, we also observed that PP2A expression was increased during the apoptosis of NB4-R1 cells induced by As_4_S_4_. SET exerts its potent PP2A inhibitory activity via its N-terminal sequence [38]. PP2A represents an abundant class of structurally complex Ser/Thr phosphatases in mammalian cells, which maintains cell homoeostasis by counteracting most of the kinase-driven intracellular signaling pathways, have been shown to be genetically altered or functionally inactivated in many solid cancers and leukaemias, and is therefore a tumor suppressor [39], [40]. Suppression of SET/I2PP2A by short hairpin RNAs was observed and resulted in an increase of PP2A activity and a reduction in BCR/ABL leukemogenesis in vivo [41]. Therefore, As_4_S_4_ inhibitions of SET expression may result in the activation of PP2A, which may lead to the dephosphorylation of PP2A target genes, thereby culminating in the induction of apoptosis in NB4-R1 cells.

APL is characterized by a chromosomal translocation, which results in the fusion gene between the genes of promyelocytic leukemia (PML) and retinoic acid receptor α (RAR α), and finally produces a fusion protein, PML-RARα. The PML-RARα protein is able to form homo/heterodimers that sequestrate PML proteins in a large protein complex. This result in the disruption of RA signal pathway and the repression of the transcriptional expression of target genes that are essential for granulocytic differentiation and apoptosis [4], [5]. Previous studies showed that As_4_S_4_ was able to decrease positive rate of PML-RARα protein in APL patients [42], and As_4_S_4_ also makes redistribution of PML-RARα protein in leukemia cells from APL patients which is quite different from that of RA treatment [21]. Our current study revealed that As_4_S_4_ inhibits the production of PML-RARα protein in NB4-R1 cells, and this inhibition may be through the inhibition of SET, since SET down-regulation by SET-RNAi and SET-RNAi plus As_4_S_4_ significantly decreased PML-RARα protein expression, as well as SET overexpression significantly increased PML-RARα protein expression. We also confirmed that the inhibition of SET expression was able to promote As_4_S_4_ induced apoptosis, while SET over-expression was able to inhibit As_4_S_4_ induced apoptosis in NB4-R1 cells. These results indicate that SET maybe an upstream gene of PML-RARα and may possibly be involved in PML-RARα down-regulation and promotes As_4_S_4_ induced apoptosis in NB4-R1 cells.

In conclusion, As_4_S_4_ was determined that it is able to inhibit the growth and induce apoptotic cells in retinoic acid (RA)-resistant NB4-R1 cells. Eight proteins including oncoprotein SET/TAF-1β were significantly changed (more than 3-fold changes) in As_4_S_4_ treated NB4-R1 cells. This study identified SET/TAF-1β is a critical gene in As_4_S_4_ induced apoptosis in NB4-R1 cells and may be a potential novel effective therapeutic target for RA-resistant APL.
