# Expression of Concern: Arsenic Sulfide Promotes Apoptosis in Retinoid Acid Resistant Human Acute Promyelocytic Leukemic NB4-R1 Cells through Downregulation of SET Protein

**DOI:** 10.1371/journal.pone.0099923

**Published:** 2014-06-20

**Authors:** The PLOS ONE Editors

The authors of the article "Arsenic Sulfide Promotes Apoptosis in Retinoid Acid Resistant Human Acute Promyelocytic Leukemic NB4-R1 Cells through Downregulation of SET Protein" recently requested the retraction of this publication. During the evaluation of this request, the *PLOS ONE* editors have identified a number of concerns about the article:

- The article reports the same work as the publication below in *Tumor Biology*, published three days after the *PLOS ONE* article:

Tetra-arsenic tetra-sulfide (As_4_S_4_) promotes apoptosis in retinoid acid -resistant human acute promyelocytic leukemic NB4-R1 cells through downregulation of SET protein

Liu Y, He P, Liu F, Zhou N, Cheng X, Shi L, Zhu H, Zhao J, Wang Y, Zhang M.

Tumour Biol. 2014 Apr;35(4):3421-30. doi: 10.1007/s13277-013-1452-1.

- The *PLOS ONE* article includes an additional author as first author, Yuwang Tian, who has not been included in the author list of the publication in *Tumor Biology*. The first author was added to the author list of the *PLOS ONE* article after the manuscript had been editorially accepted and before its publication.

We have followed up with the authors in relation to these concerns and they have indicated that the manuscript was submitted to *PLOS ONE* without their knowledge by a contractor hired to edit the language and that the first author Yuwang Tian is not a member of their research team.

In the light of the concerns about the duplicate publication of the same work and the difference in the author list for the two articles, the *PLOS ONE* editors have contacted the Xi'an Jiaotong University to request an institutional investigation.

The *PLOS ONE* editors are issuing this Expression of Concern to alert readers of the concerns about this publication. We will take further steps in relation to this article as necessary according to the outcome of the institutional investigation.
